# The spike protein of SARS-CoV-2 variant A.30 is heavily mutated and evades vaccine-induced antibodies with high efficiency

**DOI:** 10.1038/s41423-021-00779-5

**Published:** 2021-10-25

**Authors:** Prerna Arora, Cheila Rocha, Amy Kempf, Inga Nehlmeier, Luise Graichen, Martin S. Winkler, Martin Lier, Sebastian Schulz, Hans-Martin Jäck, Anne Cossmann, Metodi V. Stankov, Georg M. N. Behrens, Stefan Pöhlmann, Markus Hoffmann

**Affiliations:** 1 Infection Biology Unit, German Primate Center, Kellnerweg 4, 37077 Göttingen, Germany; 2 Faculty of Biology and Psychology, Georg-August-University Göttingen, Wilhelmsplatz 1, 37073 Göttingen, Germany; 3 Department of Anesthesiology, University of Göttingen Medical Center, Georg-August University of Göttingen, Robert-Koch-Straße 40, 37075 Göttingen, Germany; 4 Division of Molecular Immunology, Department of Internal Medicine 3, Friedrich-Alexander University of Erlangen-Nürnberg, Glückstraße 6, 91054 Erlangen, Germany; 5 Department for Rheumatology and Immunology, Hannover Medical School, Carl-Neuberg-Straße 1, 30625 Hannover, Germany

**Keywords:** Antibodies, Infection

The COVID-19 pandemic, caused by SARS-CoV-2, continues to rage in many countries, straining health systems and economies. Vaccines protect against severe disease and death and are considered central to ending the pandemic. COVID-19 vaccines (and SARS-CoV-2 infection) elicit antibodies that are directed against the viral spike (S) protein and neutralize the virus. However, the emergence of SARS-CoV-2 variants with S protein mutations that confer resistance to neutralization might compromise vaccine efficacy [1]. Furthermore, emerging viral variants with enhanced transmissibility, likely due to altered virus-host cell interactions, might rapidly spread globally. Therefore, it is important to investigate whether emerging SARS-CoV-2 variants exhibit altered host cell interactions and resistance against antibody-mediated neutralization.

We investigated host cell entry and antibody-mediated neutralization of the variant A.30 (also termed A.VOI.V2), which was detected in several patients in Angola and Sweden in spring 2021 and likely originated in Tanzania [2]. For comparison, we analyzed the Beta (B.1.351) and Eta (B.1.525) variants. These two variants were first detected in Africa, and the Beta variant, which is considered a variant of concern (VOC), shows the highest level of neutralization resistance among SARS-CoV-2 VOCs [3, 4]. Compared to the S protein of SARS-CoV-2 B.1, which circulated in the early phase of the pandemic, the S protein of the A.30 variant contains 10 amino acid substitutions and five deletions (Fig. 1a and Supplementary information, Fig. S1a). All deletions along with four substitutions are found in the N-terminal domain of the surface unit S1, which harbors an antigenic supersite that is targeted by most neutralizing antibodies not directed against the receptor-binding domain (RBD) [5]. In addition, three mutations are located inside the RBD, which binds to the cellular receptor ACE2 and constitutes the main target of neutralizing antibodies (Fig. 1a). Two of these mutations, T478R and E484K, are located close to the ACE2 binding site (Supplementary information, Fig. S1a), and E484K is known to reduce susceptibility to antibody-mediated neutralization. Finally, two mutations are located close to the S1/S2 cleavage site, and one mutation is found in the transmembrane unit S2, which facilitates fusion of the viral envelope with cellular membranes (Fig. 1a).

**Fig. 1:**
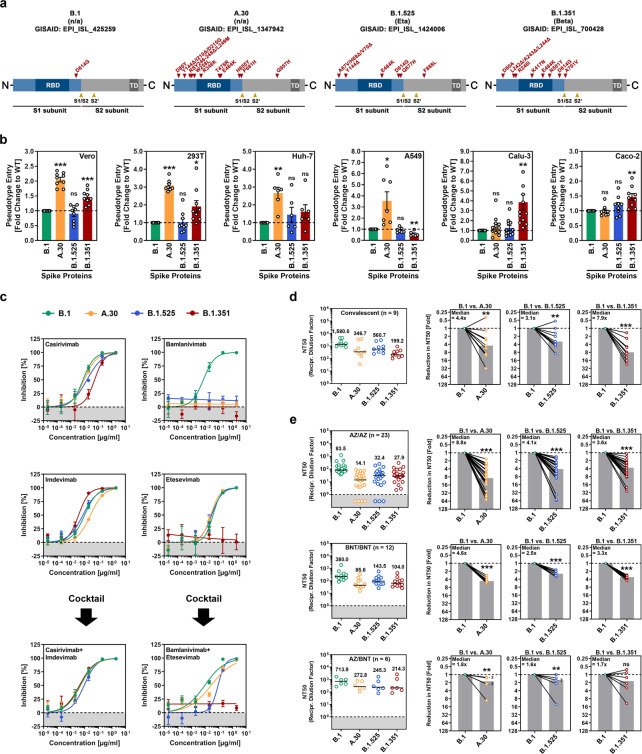
SARS-CoV-2 A.30 enters certain cell lines with increased efficiency and evades antibody-mediated neutralization. **a** Schematic overview and domain organization of the SARS-CoV-2 S proteins studied. Abbreviations: RBD, receptor-binding domain; TD, transmembrane domain. **b** Pseudotyped particles bearing the indicated S proteins were inoculated onto different cell lines, and transduction efficiency was quantified by measuring virus-encoded luciferase activity in cell lysates at 16–18 h postinoculation. Presented are the average (mean) data from six to 12 biological replicates (each conducted with technical quadruplicates) for which transduction was normalized against SARS-CoV-2 S B.1 (= 1). Error bars indicate the standard error of the mean (SEM). The statistical significance of differences between B.1 and A.30, B.1.525, or B.1.351 was analyzed by two-tailed Student’s t-test with Welch correction (*p* > 0.05, not significant [ns]; *p* ≤ 0.05, *; p ≤ 0.01, **; *p* ≤ 0.001, ***). See also Supplemental information, Fig. S1b. **c** Neutralization of SARS-CoV-2 B.1, A.30, B.1.525, and B.1.351 by monoclonal antibodies used for COVID-19 therapy or an unrelated control antibody (Supplemental information, Fig. S1d). Pseudotyped particles were incubated for 30 min at 37 °C in the presence of increasing concentrations (0.00002, 0.0002, 0.002, 0.02, 0.2, 2 µg/ml) of the indicated monoclonal antibodies or an unrelated control antibody before being inoculated onto Vero cells. Infection efficiency was quantified by measuring virus-encoded luciferase activity in cell lysates at 16–18 h postinoculation. Presented are average (mean) data from a single biological replicate (conducted with technical quadruplicates) for which infection was normalized against samples that did not contain antibody (= 0% inhibition). The data were confirmed in a separate independent experiment. Error bars indicate the standard deviation. **d** Neutralization of SARS-CoV-2 B.1, A.30, B.1.525, and B.1.351 by antibodies in convalescent plasma. Pseudotyped particles bearing the indicated S proteins were incubated for 30 min in the presence of different dilutions of convalescent plasma (*n* = 9). Infection efficiency was determined as described for Fig. 1b and used to calculate the plasma dilution factor leading to a 50% reduction in S protein-driven cell entry (neutralizing titer 50, NT50). Data from a total of nine convalescent plasma samples are presented (black lines and numerical values indicate the median NT50). In addition, for each plasma, the fold reduction in NT50 between SARS-CoV-2 B.1 (set as 1) and the indicated variants was calculated (gray bars indicate the median). The statistical significance of differences between the indicated groups was analyzed by a two-tailed Mann–Whitney test with a 95% confidence level (*p* > 0.05, ns; *p* ≤ 0.05, *; *p* ≤ 0.01, **; *p* ≤ 0.001, ***). **e** The experiment was performed as described in Panel d, but serum from ChAdOx1 nCoV-19/ChAdOx1 nCoV-19 (AZ/AZ; *n* = 23), BNT162b2/BNT162b2 (BNT/BNT; *n* = 12) or ChAdOx1 nCoV-19/BNT162b2 (AZ/BNT; *n* = 6)-vaccinated individuals was investigated. Numbers in the bar graphs “B.1 vs. A.30” and “B.1 vs. B.1.525” indicate the number of overlapping data points (dots)

For the analysis of viral entry into cells and its inhibition by antibodies, we employed rhabdoviral pseudotypes bearing the SARS-CoV-2 S protein, an adequate model for studying SARS-CoV-2 entry and neutralization [6]. As targets, we used the Vero and 293T (both kidney-derived), Huh-7 (liver), A549 (lung), Calu-3 (lung), and Caco-2 (colon) cell lines. B.1 entered all cell lines efficiently, and the entry efficiency of B.1.525 was comparable (Fig. 1b and Supplementary information, Fig. S1b). The entry of B.1.351 into several cell lines was slightly but significantly increased, and this phenotype was particularly robust for Calu-3 lung cells, in keeping with published results [3]. Finally, A.30 showed markedly increased efficiency regarding entry into Vero, 293 T, Huh-7, and A549 cells compared to B.1, though entry into Calu-3 and Caco-2 cells was not augmented (Fig. 1b). Testing of monoclonal antibodies directed against the S protein and used for COVID-19 therapy revealed that B.1.351 was resistant to both bamlanivimab and etesevimab, as expected [3] and that B.1.525 was resistant to bamlanivimab (Fig. 1c). A.30 was also bamlanivimab resistant but susceptible to inhibition by a cocktail of bamlanivimab and etesevimab (Fig. 1c). Furthermore, B.1.351 showed markedly reduced neutralization by antibodies induced upon infection, as expected; [3] neutralization evasion by A.30 and B.1.525 was slightly (A.30) to moderately (B.1.525) less efficient (Fig. 1d and Supplementary information, Fig. S2). Conversely, A.30 was more resistant to neutralization by antibodies induced upon homologous ChAdOx1 nCoV-19 (Vaxzevria) or BNT162b2 (Comirnaty) vaccination compared to B.1.351, but the neutralization sensitivity of B.1.525 was approximately in the same range as that of B.1.351 (Fig. 1e and Supplementary information, Fig. S2 and Table S1). Finally, all variants tested exhibited reduced and comparable evasion of antibodies induced by heterologous ChAdOx1 nCoV-19/BNT162b2 vaccination, in keeping with findings published for the Delta (B.1.617.2) variant [7].

In summary, A.30 exhibits a cell line preference not observed for other viral variants and efficiently evades neutralization by antibodies elicited by ChAdOx1 nCoV-19 or BNT162b2 vaccination. SARS-CoV-2 entry into cell lines depends on S protein activation by the cellular proteases cathepsin L or TMPRSS2 [8], and activation by the latter is thought to support viral spread in the lung. Therefore, it is noteworthy that enhanced A.30 entry was observed for cell lines with cathepsin L (Vero, 293 T, Huh-7, A549 cells)—but not TMPRSS2 (Calu-3, Caco-2)-dependent entry [8]. Thus, one could speculate that A.30 might use cathepsin L with increased efficiency and slight (but not statistically significant) resistance of A.30 against the cathepsin L inhibitor MDL 28170 supports this possibility (Supplemental information, Fig. S1c). Notably, robust entry into cell lines was combined with high resistance against antibodies induced upon ChAdOx1 nCoV-19 or BNT162b2 vaccination. Neutralization resistance exceeded that of the Beta (B.1.351) variant, which is markedly neutralization resistant in cell culture and, in comparison with the Alpha (B.1.1.7) variant, is less well inhibited by the ChAdOx1 nCoV-19 vaccine [9]. Nevertheless, heterologous ChAdOx1 nCoV-19/BNT162b2 vaccination, which was previously shown to augment neutralizing antibody responses against VOCs compared to corresponding homologous vaccinations [7, 10], might offer robust protection against the A.30 variant. Collectively, our results suggest that the SARS-CoV-2 variant A.30 can evade control by vaccine-induced antibodies and might show an increased capacity to enter cells in a cathepsin L-dependent manner, which might particularly aid in the extrapulmonary spread. As a consequence, the potential spread of the A.30 variant warrants close monitoring and rapid installment of countermeasures.

## Supplementary information

Supplemental information
