# Supplementary material for: The spike protein of SARS-CoV-2 variant A.30 is heavily mutated and evades vaccine-induced antibodies with high efficiency
Source: Cell Mol Immunol. 2021 Oct 25;18(12):2673–5. doi: 10.1038/s41423-021-00779-5 (PMC8543421; doi:10.1038/s41423-021-00779-5)
Supplement: Supplementary file 1 — Supplemental information [file 41423_2021_779_MOESM1_ESM.pdf]

The spike protein of SARS-CoV-2 variant A.30 is heavily mutated and evades vaccine induced antibodies with high efficiency

Prerna Arora[1,2], Cheila Rocha[1,2], Amy Kempf[1,2], Inga Nehlmeier[1], Luise Graichen[1,2], Martin S. Winkler[3], Martin Lier[3], Sebastian Schulz[4], Hans-Martin Jäck[4], Anne Cossmann[5], Metodi V. Stankov[5], Georg M. N. Behrens[5], Stefan Pöhlmann[1,2], Markus Hoffmann[1,2]

[1]Infection Biology Unit, German Primate Center, Kellnerweg 4, 37077 Göttingen, Germany

[2]Faculty of Biology and Psychology, Georg-August-University Göttingen, Wilhelmsplatz 1, 37073 Göttingen, Germany

[3]Department of Anesthesiology, University of Göttingen Medical Center, Göttingen, Georg-August University of Göttingen, Robert-Koch-Straße 40, 37075 Göttingen, Germany

[4]Division of Molecular Immunology, Department of Internal Medicine 3, Friedrich-Alexander University of Erlangen-Nürnberg, Glückstraße 6, 91054 Erlangen, Germany

[5]Department for Rheumatology and Immunology, Hannover Medical School, Carl-Neuberg-Straße 1, 30625 Hannover, Germany

Correspondence: Markus Hoffmann (mhoffmann@dpz.eu) or Stefan Pöhlmann (spoehlmann@dpz.eu)

Methods and materials

Cell culture

293T (human, female, kidney; ACC-635, DSMZ; RRID: CVCL_0063), Vero cells (African green monkey kidney, female, kidney; CRL-1586, ATCC; RRID: CVCL_0574, kindly provided by Andrea Maisner) and Huh-7 (human, male, liver; JCRB Cat# JCRB0403; RRID: CVCL_0336, kindly provided by Thomas Pietschmann), were cultured in Dulbecco's modified Eagle medium (PAN-Biotech). A549 cells (human, male, lung; CRM-CCL-185, ATCC; RRID: CVCL_0023; kindly provided by Georg Herrler) were cultivated in DMEM/F-12 Medium with Nutrient Mix (Thermo Fisher Scientific). Additionally, Calu-3 (human, male, lung; HTB-55, ATCC; RRID: CVCL_0609, kindly provided by Stephan Ludwig) and Caco-2 cells (human, male, colon; HTB-37, ATCC, RRID: CVCL_0025) were cultured in minimum essential medium (GIBCO). Media were supplemented with 10% FBS (Biochrom) and 1% pen/strep (PAA). Calu-3 and Caco-2 cells further received 1x non-essential amino acid solution (from 100x stock, PAA) and 1 mM sodium pyruvate (GIBCO). All cell lines were incubated at 37 °C in a humidified atmosphere containing 5% CO_2. Cell lines were validated by STR-typing, amplification and sequencing of a fragment of the cytochrome c oxidase gene, microscopic examination and/or according to their growth characteristics. Furthermore, all cell lines were routinely tested for mycoplasma contamination.

Sequence analysis and protein models

The S protein sequences of SARS-CoV-2 S B.1 (GISAID Accession ID: EPI_ISL_425259) and B.1.351 variants (GISAID Accession ID: EPI_ISL_700428) have been described previously [1-3]. The S protein sequences of SARS-CoV-2 S variants A.30 (GISAID Accession ID:

EPI_ISL_1347942) and B.1.525 (GISAID Accession ID: EPI_ISL_1424006) were obtained from the GISAID (global initiative on sharing all influenza data) database (https://www.gisaid.org/). Protein models were generated employing the YASARA software (http://www.yasara.org/index.html) and are based on a template that was constructed by modelling the SARS-2 S sequence on PDB: 6XR8 [4] using the SWISS-MODEL online tool (https://swissmodel.expasy.org)

Expression plasmids

Plasmids pCAGGS-DsRed, pCAGGS-VSV-G (vesicular stomatitis virus glycoprotein), pCG1-SARS-CoV-2 S B.1 (codon optimized, contains C-terminal truncation of the last 18 amino acid) and pCG1-SARS-CoV-2 S B.1.351 have been previously described [1-3]. In order to generate the expression vector for the S protein of SARS-CoV-2 variants A.30 and B.1.525, the respective mutations were inserted into the expression plasmid for the wildtype SARS-CoV-2 S sequence (Wuhan/Hu-1/2019 isolate, GISAID Accession ID: EPI_ISL_406798, codon-optimized) [3] by splice overlap PCR. The resulting open reading frames were further inserted into the pCG1 vector (kindly provided by Roberto Cattaneo, Mayo Clinic College of Medicine, Rochester, MN, USA), using BamHI and XbaI restriction enzymes. The integrity of all sequences was confirmed by sequence analysis using a commercial sequencing service (Microsynth SeqLab). Specific details on the cloning procedure can be obtained upon request. Transfection of 293T cells was carried out by the calcium-phosphate precipitation method.

Production of pseudotype particles and transduction of target cells

Rhabdoviral pseudotypes bearing SARS-CoV-2 spike protein were generated according to an established protocol [5]. Briefly, 293T cells were transfected with expression plasmids encoding S protein, VSV-G or DsRed (control). At 24 h posttransfection, cells were inoculated with a replication-deficient vesicular stomatitis virus that lacks the genetic information for VSV-G and instead codes for two reporter proteins, enhanced green fluorescent protein and firefly luciferase (FLuc), VSV∗ΔG-FLuc (kindly provided by Gert Zimmer) at a multiplicity of infection of 3. Following 1 h of incubation at 37 °C, the inoculum was removed and cells were washed with phosphate-buffered saline (PBS). Subsequently, cells received culture medium containing anti-VSV-G antibody (culture supernatant from I1-hybridoma cells; ATCC no. CRL-2700; except for cells expressing VSV-G, which received only medium) in order to neutralize residual input virus. After 16-18h, the culture supernatant was harvested, clarified from cellular debris by centrifugation at 4,000 x g, 10 min, aliquoted and stored at -80 °C. For transduction experiments, target cells were seeded in 96-well plates and inoculated with equal volumes of pseudotype particles. The transduction efficiency was evaluated at 16-18 h post transduction. For this, cells were lysed in PBS containing 0.5% triton X-100 (Carl Roth) for 30 min at RT. Afterwards, cell lysates were transferred into white 96-well plates and mixed with luciferase substrate (Beetle- Juice, PJK) before luminescence was recorded using a Hidex Sense Plate luminometer (Hidex).

Treatment of cells with MDL 28170 inhibitor

In order to block activity of cathepsin L, MDL 28170 (Sigma-Aldrich) [6] was used. MDL 28170 was reconstituted in dimethyl sulfoxide (DMSO) and prepared as stock solution of 50 mM concentration. Vero cells were treated with MDL 28170 for 1h at 37 °C before transduction. Cells treated with DMSO served as a control.

Serum and plasma samples

Before analysis, all serum and plasma samples were heat-inactivated at 56 °C for 30 min. Further, all plasma/serum samples were pre-screened for their ability to neutralize transduction of Vero cells by pseudotype particles bearing SARS-CoV-2 S B.1.

Convalescent plasma was obtained from COVID-19 patients treated at the intensive care unit of the University Medicine Göttingen (UMG) under approval given by the ethic committee of the UMG (SeptImmun Study 25/4/19 Ü). Patient details can be found elsewhere [7]. In addition, sera from individuals vaccinated with ChAdOx1 nCoV-19/ChAdOx1 nCoV-19, BNT162b2/BNT162b2 or ChAdOx1 nCoV-19/BNT162b2 were collected 27-204 days after receiving the second dose under the approval given by the ethic committee of the UMG (reference number: 8/9/20). Details can be found in Table S1.

Neutralization assay

For neutralization experiments, S protein bearing pseudotype particles were pre-incubated for 30 min at 37 °C with different concentrations of Casirivimab, Imdevimab, Bamlanivimab, Etesevimab, Casirivimab + Imdevimab, Bamlanivimab + Etesevimab or unrelated control IgG (2, 0.2, 0.02, 0.002, 0.0002, 0.00002 µg/ml). Alternatively, pseudotype particles were pre-incubated with different dilutions (dilution range: 1:6.25 to 1:25,600) of convalescent plasma or serum from vaccinated individuals. Following incubation, mixtures were inoculated onto Vero cells with particles incubated only with medium serving as controls. Transduction efficiency was determined at 16-18 h postinoculation as described above.

Statistical analysis

The results on S protein-driven cell entry represent average (mean) data acquired from six to twelve biological replicates, each conducted with four technical replicates. The transduction was normalized against SARS-CoV-2 S B.1 (= 1). Alternatively, transduction was normalized against the background signal (luminescence measured for cells inoculated with particles bearing no viral glycoprotein; set as 1). For inhibition experiments using MDL 28170, presented are the average (mean) data from three biological replicates (conducted with technical quadruplicates) for which transduction was normalized against samples that did receive DMSO instead of inhibitor (= 0% inhibition). The results on neutralization of spike protein-driven cell entry by monoclonal antibodies and IgG represent average (mean) data from a single biological replicate (conducted with technical quadruplicates) for which transduction was normalized against samples that did not contain any antibody (= 0% inhibition). The data were confirmed in a separate independent experiment. The results on neutralization of spike protein-driven cell entry by convalescent plasma or serum from vaccinated individuals are based on a single experiment, which was conducted with technical quadruplicates. For data normalization, the plasma/serum dilution factor that leads to 50% reduction in S protein-driven cell entry (neutralizing titer 50, NT50) was calculated. In addition, for each plasma/serum the fold reduction in NT50 between SARS-CoV 2 B.1 (set as 1) and the indicated variants was calculated.

Error bars are defined as either standard deviation (SD) or standard error of the mean (SEM). Data were analyzed using Microsoft Excel (as part of the Microsoft Office software package, version 2019, Microsoft Corporation) and GraphPad Prism 8 version 8.4.3 (GraphPad Software). Statistical significance was analyzed by two-tailed Student's t-test with Welch correction (pseudotype entry,

MDL inhibition) or two-tailed Mann-Whitney test with 95% confidence level (neutralization). Only p-values of 0.05 or lower were considered statistically significant ($p > 0.05$, not significant [ns]; $p \leq 0.05$, *; $p \leq 0.01$, **; $p \leq 0.001$, ***). Details on the statistical test and the error bars can be found in the figure legends.

REFERENCES

1 Hoffmann, M. *et al.* SARS-CoV-2 variants B.1.351 and P.1 escape from neutralizing antibodies. *Cell*, doi:10.1016/j.cell.2021.03.036 (2021).

2 Hoffmann, M. *et al.* SARS-CoV-2 variant B.1.617 is resistant to Bamlanivimab and evades antibodies induced by infection and vaccination. *bioRxiv*, 2021.2005.2004.442663, doi:10.1101/2021.05.04.442663 (2021).

3 Hoffmann, M. *et al.* SARS-CoV-2 Cell Entry Depends on ACE2 and TMPRSS2 and Is Blocked by a Clinically Proven Protease Inhibitor. *Cell* **181**, 271-280 e278, doi:10.1016/j.cell.2020.02.052 (2020).

4 Cai, Y. *et al.* Distinct conformational states of SARS-CoV-2 spike protein. *Science* **369**, 1586-1592, doi:10.1126/science.abd4251 (2020).

5 Berger Rentsch, M. & Zimmer, G. A vesicular stomatitis virus replicon-based bioassay for the rapid and sensitive determination of multi-species type I interferon. *PLoS One* **6**, e25858, doi:10.1371/journal.pone.0025858 (2011).

6 Simmons, G. *et al.* Inhibitors of cathepsin L prevent severe acute respiratory syndrome coronavirus entry. *Proc Natl Acad Sci U S A* **102**, 11876-11881, doi:10.1073/pnas.0505577102 (2005).

7 Hoffmann, M. *et al.* SARS-CoV-2 variants B.1.351 and P.1 escape from neutralizing antibodies. *Cell* **184**, 2384-2393 e2312, doi:10.1016/j.cell.2021.03.036 (2021).

Table S1: Vaccinated patient data.

ID	Age group (y)	Gender	1st vaccination	2nd vaccination	Time between 1st & 2nd vaccination (d)	Time since 2nd vaccination (d)
**6365**	25-34	F	ChAdOx1-SARS-COV-2 (AZ)	ChAdOx1-SARS-COV-2 (AZ)	74	42
**6499**	35-44	F	ChAdOx1-SARS-COV-2 (AZ)	ChAdOx1-SARS-COV-2 (AZ)	75	28
**6205**	55-64	F	ChAdOx1-SARS-COV-2 (AZ)	ChAdOx1-SARS-COV-2 (AZ)	71	27
**6230**	35-44	F	ChAdOx1-SARS-COV-2 (AZ)	ChAdOx1-SARS-COV-2 (AZ)	71	27
**6239**	55-64	F	ChAdOx1-SARS-COV-2 (AZ)	ChAdOx1-SARS-COV-2 (AZ)	75	27
**6249**	55-64	F	ChAdOx1-SARS-COV-2 (AZ)	ChAdOx1-SARS-COV-2 (AZ)	82	28
**6278**	45-54	F	ChAdOx1-SARS-COV-2 (AZ)	ChAdOx1-SARS-COV-2 (AZ)	82	28
**6297**	45-54	F	ChAdOx1-SARS-COV-2 (AZ)	ChAdOx1-SARS-COV-2 (AZ)	81	29
**6351**	25-34	F	ChAdOx1-SARS-COV-2 (AZ)	ChAdOx1-SARS-COV-2 (AZ)	74	29
**6243**	55-64	F	ChAdOx1-SARS-COV-2 (AZ)	ChAdOx1-SARS-COV-2 (AZ)	79	30
**6262**	55-64	F	ChAdOx1-SARS-COV-2 (AZ)	ChAdOx1-SARS-COV-2 (AZ)	71	30
**6272**	55-64	F	ChAdOx1-SARS-COV-2 (AZ)	ChAdOx1-SARS-COV-2 (AZ)	80	32
**6276**	55-64	F	ChAdOx1-SARS-COV-2 (AZ)	ChAdOx1-SARS-COV-2 (AZ)	79	32
**6235**	25-34	F	ChAdOx1-SARS-COV-2 (AZ)	ChAdOx1-SARS-COV-2 (AZ)	69	33
**6321**	25-34	F	ChAdOx1-SARS-COV-2 (AZ)	ChAdOx1-SARS-COV-2 (AZ)	77	33
**6358**	25-34	F	ChAdOx1-SARS-COV-2 (AZ)	ChAdOx1-SARS-COV-2 (AZ)	92	33
**6236**	55-64	F	ChAdOx1-SARS-COV-2 (AZ)	ChAdOx1-SARS-COV-2 (AZ)	76	34
**6251**	55-64	F	ChAdOx1-SARS-COV-2 (AZ)	ChAdOx1-SARS-COV-2 (AZ)	76	34
**6283**	45-54	M	ChAdOx1-SARS-COV-2 (AZ)	ChAdOx1-SARS-COV-2 (AZ)	76	34
**6294**	35-44	F	ChAdOx1-SARS-COV-2 (AZ)	ChAdOx1-SARS-COV-2 (AZ)	76	34
**L2**	25-34	M	ChAdOx1-SARS-COV-2 (AZ)	BNT162b2 (BNT)	80	65
**L6**	25-34	F	ChAdOx1-SARS-COV-2 (AZ)	BNT162b2 (BNT)	80	65
**L15**	25-34	M	ChAdOx1-SARS-COV-2 (AZ)	BNT162b2 (BNT)	70	92
**L17**	18-24	F	ChAdOx1-SARS-COV-2 (AZ)	ChAdOx1-SARS-COV-2 (AZ)	77	84
**L18**	18-24	F	ChAdOx1-SARS-COV-2 (AZ)	ChAdOx1-SARS-COV-2 (AZ)	84	77

**L20**	18-24	F	ChAdOx1-SARS-COV-2 (AZ)	BNT162b2 (BNT)	84	81
**L23**	25-34	F	ChAdOx1-SARS-COV-2 (AZ)	BNT162b2 (BNT)	84	98
**L24**	18-24	F	ChAdOx1-SARS-COV-2 (AZ)	BNT162b2 (BNT)	84	98
**L27**	35-44	M	ChAdOx1-SARS-COV-2 (AZ)	ChAdOx1-SARS-COV-2 (AZ)	73	77

Figure S1

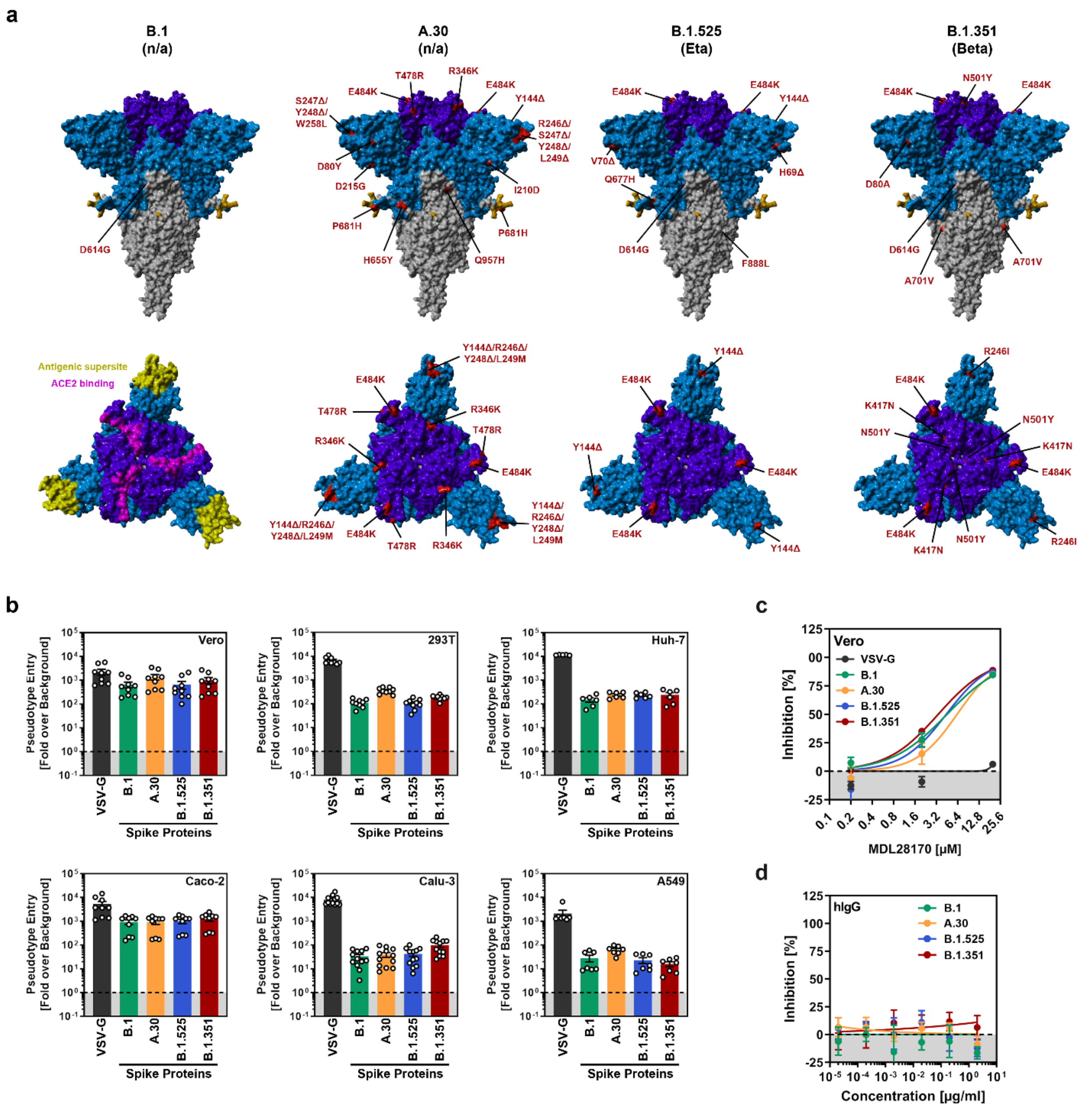

Figure S1. Location of mutations in A.30 and B.1.525 spike and functional analysis of the spike proteins. a Location of the mutations (compared to the S protein of Wuhan-Hu-1 isolate, GISAID Accession ID: EPI_ISL_406798) in the context of the trimeric spike protein (Color code: light blue, S1 subunit with RBD in dark blue; gray, S2 subunit; orange, S1/S2 and S2' cleavage sites; red, mutated amino acid residues; yellow, antigenic supersite; pink, RBD residues that interact with ACE2). **b** Transduction data presented in figure 1b normalized against the assay background. The experiment was performed as described in the legend of figure 1b with the difference that transduction was normalized against signals obtained from cells inoculated with particles bearing no viral glycoprotein (background, set as 1). Further, transduction by particles bearing VSV-G is shown. Error bars indicate the standard error of the mean (SEM). **c** Evidence that A.30 may use cathepsin L with slightly increased efficiency. Vero cells

were incubated for 1 h in the presence of different concentrations (0, 0.2, 2, 20 µM) of cathepsin L inhibitor MDL 28170 before particles bearing the indicated S proteins (or VSV-G) were added. Transduction efficiency was determined as described for figure 1b. Presented are the average (mean) data from three biological replicates (conducted with technical quadruplicates) for which transduction was normalized against samples that did receive DMSO instead of inhibitor (= 0% inhibition). Error bars indicate the SEM. **d** The neutralization experiment described in the legend of figure 1c was conducted using a non-neutralizing control antibody (hIgG).

Figure S2

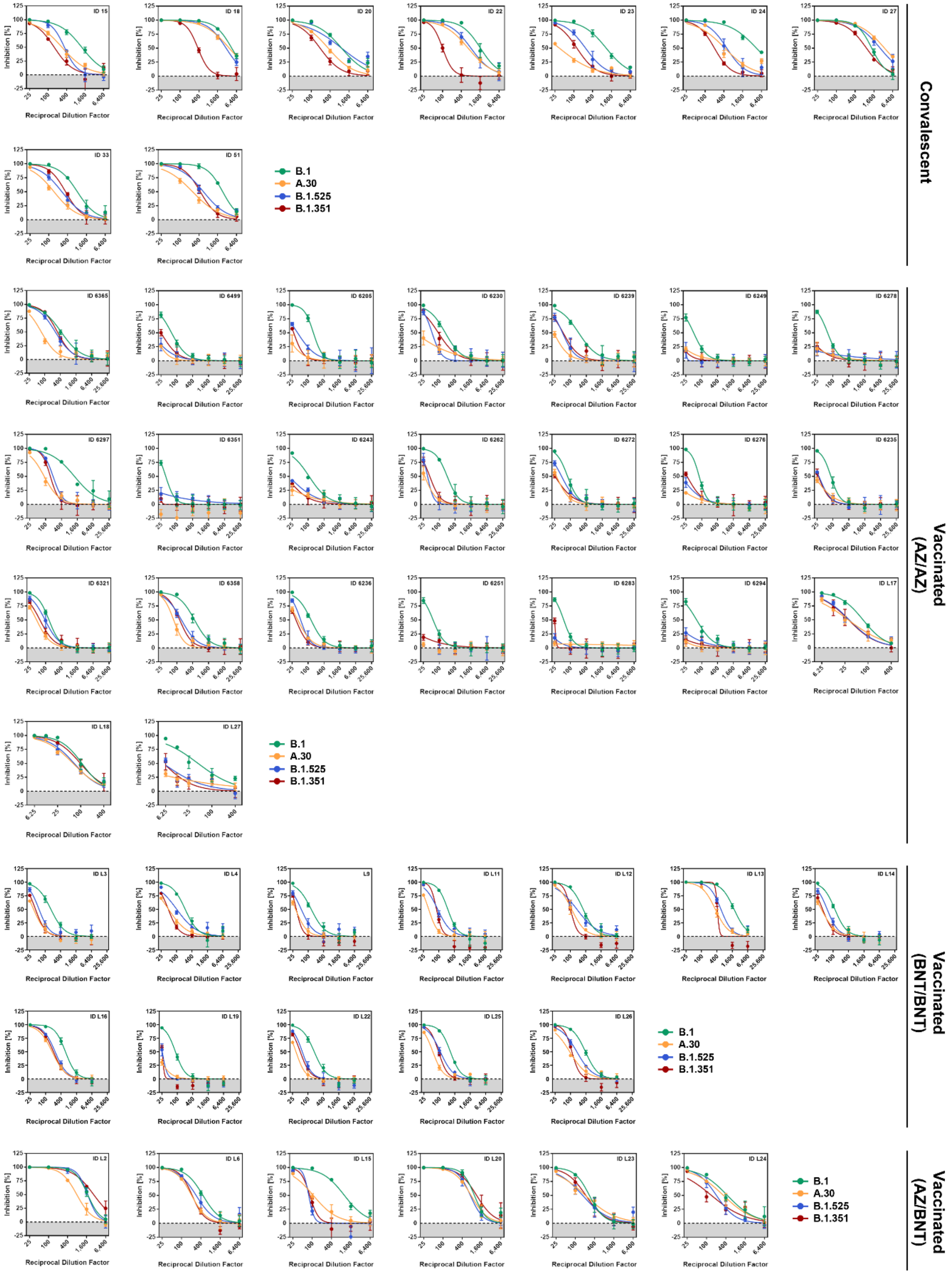
Convalescent
Vaccinated (AZ/AZ)
Vaccinated (BNT/BNT)
Vaccinated (AZ/BNT)
B.1
A.30
B.1.525
B.1.351
Inhibition [%]
Reciprocal Dilution Factor
ID 15
ID 18
ID 20
ID 22
ID 23
ID 24
ID 27
ID 33
ID 51
ID 6365
ID 6499
ID 6205
ID 6230
ID 6239
ID 6249
ID 6278
ID 6297
ID 6351
ID 6243
ID 6262
ID 6272
ID 6276
ID 6235
ID 6321
ID 6358
ID 6236
ID 6251
ID 6283
ID 6294
ID L17
ID L18
ID L27
ID L3
ID L4
L9
ID L11
ID L12
ID L13
ID L14
ID L16
ID L19
ID L22
ID L25
ID L26
ID L2
ID L6
ID L15
ID L20
ID L23
ID L24

Figure S2: Individual neutralization data. Presented are the individual neutralization results corresponding to the data presented in Fig. 1d (convalescent) and e (vaccinated). All data show the mean values of four technical replicates with error bars indicating the standard deviation. The curves were calculated based on a non-linear regression model with variable slope. Abbreviations: AZ/AZ, ChAdOx1 nCoV-19/ChAdOx1 nCoV-19; BNT/BNT, BNT162b2/ BNT162b2; AZ/BNT, ChAdOx1 nCoV-19/BNT162b2.
